# Level of attitude, knowledge and practice of nurses toward postoperative pain management, cross-sectional study

**DOI:** 10.1016/j.amsu.2022.104902

**Published:** 2022-11-17

**Authors:** Zenebe Bekele Teshome, Yibeltal Aychew, Wubshet Mitiku, Beyene Guta

**Affiliations:** Department of Anesthesia, College of Medicine and Health Sciences, Ambo University, Ambo, Ethiopia

**Keywords:** Attitude, Knowledge, Practice, Nurse and postoperative pain management, IASP, International Association for the Study of Pain, POP, Post Operative Pain, SPSS, Statistical SoftWare for Social Science

## Abstract

**Background:**

Patients still experience unnecessary pain in many hospitals, especially after surgery, despite increasing awareness of pain management in many healthcare settings. Unrelieved postoperative pain has been shown to increase the incidence of postoperative complications (such as atelectasis, pneumonia, thromboembolism, and impaired immune function. Little known and not known, evidence to understand gaps in nurses' attitudes and practices regarding postoperative pain management in our hospital.

**Methods:**

A descriptive cross-sectional study was performed with 144 nurses A systematic random sampling technique was used to select study participants. Data were collected using a self-administered and verified structured questionnaire; Data were entered and analyzed using SPSS software version 24. Descriptive results are presented by frequency, percentage, mean, bar graph, and pie chart.

**Results:**

Overall results from 144 study participants showed that nurses had good knowledge 78 (54.2%), favorable attitudes 67 (60.4%), and practice 81 (56). %) on pain management after surgery. In terms of nurse training, 60 (41.7%) have a bachelor's degree holders and only 34 (23.6%) nurses are trained in postoperative pain management.

**Conclusions:**

In this study, the nurses' overall knowledge of postoperative management was good, with favorable attitudes and good practices. But the level of knowledge, attitude, and practice according to the research are only average; therefore, it will make it possible to recommends to the responsible authorities of universities, hospitals, and nurses to organize continuing education.

## Highlights

• Nurses are key professional for postoperative pain management.
• The level of knowledge, attitude and practice in this study was around mean score.
• Practice of training and availability of postoperative pain management protocol was in adequate.

## 1 Introduction

The International Association for the Study of Pain (IASP, 1994) defines pain as an unpleasant sensory and emotional experience associated with actual or potential tissue damage [1]. According to a study by Ayla Yava et al., in Turkey, patients still have pain in some hospitals, especially after surgery, despite the awareness of pain management in some medical facilities today increasing. The nurse plays a much-needed role in effective pain management from surgery, which is why it is her responsibility to assess pain and intervene promptly. Several disciplines are involved in pain management; but the nurse plays an important role in assessment, pain relief, and analysis. Pain expertise can be common, depending on the individual's interpretation. It manifests itself in many ways that are difficult to accurately assess [[2], [3], [4], [5]]. The incidence of surgical pain (POP) is between 47 and 100%. Studies have consistently confirmed over the past 3-4 decades that 20–80% of surgical patients have untreated pain and that pain is classified as a serious public health condition in every developed country rather than in any developed country. The developing countries, nurse's attitudes and data regarding pain management have important implications for patient treatment and care [4,5,[13], [14], [15], [16], [17]]. The importance of pain management during surgery is undeniable. Over the past 20 years, intractable surgical pain has been shown to increase the incidence of surgical complications (such as pathology, pneumonia, etc.), embolism, impaired immune performance, and prolonged hospital stay) and risk. develop chronic pain during surgery, despite continued cutting-edge analysis unquestionably in pain control, the pain has not been adequately relieved, resulting in patient dissatisfaction, high complications, and slow wound healing, especially in developing countries, which are often hampered by lack of hospital funding, lack of education and training programs, and lack of data on a wide range of options, drug selection, technical skills, and attitudes, persistent negative attitudes toward pain-relieving treatments, especially opioids, and poor health care systems that propagate a treatment culture that does not exist or is suboptimal [[18], [19], [20], [21], [22], [23], [24]]. According to my research, there is no data on nurses' knowledge, attitudes, or practices in postoperative pain management. Therefore, this study aimed to evaluate the amount of evidence, nurses' attitudes, and practices in surgical pain management in our low-resource setting.

## 2 Methods and materials

### 2.1 Study area and period

The study was conducted at Ambo University Referral Hospital, which is located in the West Shoa, Oromia region of Ethiopia, about 114 Km from Addis Ababa. The hospital is serving more than 2 million people in the catchment area in all four major departments and other units since 2017 GC. This Zone has a total population of 2,058,676, of whom 1,028,501 are men and 1,030,175 women; with an area of 14,788.78 square kilometers, West Shoa has a population density of 139.21. The study was conducted from December 10/2014 EC to January 10/2014 EC. Registered in the Research Registry and UIN = 7800 and the methodology in this study has been reported according to STROSS guidelines 2021 [6,30].

### 2.2 Study design

An institutional-based descriptive cross-sectional study.

### 2.3 Population

#### 2.3.1 Source population

All nurse staff working in the study area.

### 2.4 Study population

Nurses working in postoperative care who met incursion criteria in the study period.

### 2.5 Eligibility criteria

#### 2.5.1 Inclusion

All Nurses who are working in postoperative care.

#### 2.5.2 Exclusion

✓ Nurses who don't found during data collection; critically sick, annual leave nurses are excluded during data collection time.

### 2.6 Sample size determination

The sample size was calculated by using the single population proportion formula, for knowledge, Attitude, and practice separately; finally, the largest sample size was taken. The prevalence of knowledge was found at 54% in Asela Zonal Hospital, Ethiopia [7,8]. The confidence interval of 95% and margin of error of 0.05

$$n=\frac{{\left(z\alpha /2\right)}^{2}pq}{d^{2}}$$

n = 384 using correction formula sample size was 131 by adding 10% non-response rate our final sample size was 144.

### 2.7 Sampling technique

144 registered nurses were recruited from various surgical units of the hospital. A complete 144 respondents completed their questionnaire out of the eligible 144 (100% response rate). The study utilized the systematic random sampling technique in recruiting the nurses into the study. The nurses were approached at the time of the study at the chosen units, explained the aim of the study, and requested for his or their participation.

### 2.8 Study variables

#### 2.8.1 Dependent variables

Knowledge, attitude and practice.

### 2.9 Operational definition

**Knowledge**: means the nurses’ perception and understanding of post-operative pain management based on experience. This is categorized as good knowledge and poor knowledge based on the mean.

**Good knowledge** is the knowledge status of nurses when they score more than or equal to the mean.

**Poor knowledge** is the knowledge status of nurses when they score less than the mean.

**Attitude:** refer to the nurses' behavior and way of acting toward effective pain management. This is categorized as a favorable attitude and an unfavorable attitude.

**A favorable attitude** is the category of nurses when they score more than or equal to the mean value.

**Unfavorable attitude:** is the category of nurses when they score less than the mean.

**Practice:** This means the nurses’ skills in post-operative pain management are based on their experience.

**A good practice** is the practice status of nurses when they score more than or equal to the mean.

**Poor practice** is the practice status of nurses when they score less than the mean.

### 2.10 Data collection tool and procedures

A structured self-administered questionnaire was used to collect information from study participants. This tool has been developed and adjusted after an extensive literature review. It was drafted in English and the questionnaire also included four sections covering the nurses' socio-demographic status; their knowledge of POP assessment and management; their attitudes towards the assessment and POP management, and POP assessment and management practices. Data were collected by third and fourth-year anaesthesia students. The scientist organized a 3-day training course for the data collectors to familiarize them with the data collection tool. The scientist supports and coordinates the information collectors. Data is collected from selected professionals in each department. The scientist was responsible for coordinating the medical professionals and discussing the purpose of the study. Then, to support their willingness to participate, a questionnaire was distributed and instructions on how to complete the questionnaire, and an explanation for any difficulties.

### 2.11 Data processing and analysis

Data were verified, coded, and analyzed by SPSS version 24 software. Descriptive results were presented by frequency, percentage, bar graph, pie chart and mean. Finally, using international and national data, the results were compared and discussed and the conclusion and recommendation were forwarded.

### 2.12 Ethical consideration

The data was collected after a formal letter of cooperation is sent from Ambo University College of medicine and health science student Research program office and Anaesthesia department. Moreover, individuals were asked to fill out questionnaires after informed verbal consent was obtained.

## 3 Result

### 3.1 Part one: socio-demographic characteristics of respondents

In this study, a total of 144 participants were involved; this makes a response rate of 100%. The mean age of the respondents was 27 yrs with ±3.13SD. Regarding gender 60(41.7%) were female. Concerning educational qualifications 60(41.7%) were BSc and 115(79.9%) participants had more than one year of experience. Only 34(23.6%) of nurses have been trained in postoperative pain management. Of all participants 104 (72.2%) responds as pain management content included in their practice. Regarding pain management protocol in the participant's practice area, 83(57.6%) responds as there is no pain management protocol in their hospital. (Table 1).

**Table 1:** Socio demographic Characteristics of Respondents.

Variables	Categories	Frequency	Percentage (%)
Age	20–24 years	22	15.3
	25–29 years	89	61.8
	30–34 years	30	20.8
	35–39 years	1	0.7
	>40 years	2	1.4
Sex	Male	84	58.3
	Female	60	41.7
Duration Of Service in work	<1 years	0	0
	1–5 years	115	79.9
	>6 years	29	20.1
Educational Qualification in Nursing	Diploma	4	2.8
	BSc	131	91
	MSc	8	5.6
Experience in Month in surgical side	0–12 months	113	78.5
	13–24 months	25	17.4
	25–35 months	6	4.2
	>36 months	0	0
Marital Status	Single	42	29.2
	Married	96	66.7
	Divorced	6	4.2
	Widowed	0	0
	Separated	0	0
Religion of participants	Orthodox	34	23.6
	Muslim	19	13.2
	Protestant	85	59.0
	Catholic	0	0
	Other	6	4.2
Rank (position of the nurse)	Staff nurse	56	38.9
	Senior staff nurse	59	41.0
	Nursing officer	16	11.1
	Senior nursing officer	10	6.9
	Principal nursing officer	3	2.1
Current area of practice	Surgical	29	20.1
	Oby/Gyn	19	13.2
	OR	29	20.1
	ICU	19	13.2
	Pediatric	13	9.0
	Others	35	24.3
Training on Pain Management	Yes	34	23.6
	No	110	76.4
Is there pain management content included in nursing practices	No	40	27.8
	Yes	104	72.2
Is there any pain standard(protocol) in your hospital	No	61	42.4
	Yes	83	57.6

### 3.2 Knowledge of nurses about post-operative pain management

The mean knowledge of the respondents was 25.3 with ±2.96SD. Among study participants in this study 78(54.2%) participants have good knowledge and 66 (45.8%) had poor knowledge. Most of the participants 94(65.3%) knew that the side effects of narcotics should be observed at least 20 min after administration and 104(72.2%) of the study subject's vital signs are always reliable indicators of the intensity of patients and 64 (44.4%) participant are said Pethidine 75 mg IM is approximately equal to morphine 10 mg IM. Regarding patient pain assessment 79(54.9%) of nurses agree to adapt rating scale ranging (0) no pain at all to Ref. [10] the worst pain. Regarding pain on the moment only 77(53.5%) of participants think patients sleep with no movement postoperatively indicating that patient have no pain (Table 2).

**Table 2:** Knowledge of nurses toward post-operative pain management.

Name of the Variable	Category	Frequency	%percent
**Vital signs are always reliable indicators of the intensity of a patient's**	No	40	27.8
	Yes	104	72.2
**Because their nervous system is underdeveloped, children under two years of age have decrease pain sensitivity and limited memory of painful experiences**	No	65	45.1
	Yes	79	54.9
**Aspirin and other non-steroidal anti-inflammatory agents are not effective analgesics for acute postoperative pain**	No	63	43.8
	Yes	81	56.3
**Respiratory depression rarely occurs in patients who have been receiving stable doses of opioid over a period of months**	No	60	41.7
	Yes	84	58.3
**Combining analgesics that work by different mechanisms may result in better pain control with fewer side effects than using a single analgesic agent**	No	55	38.2
	Yes	89	61.8
**Pethidine 75 mg IM is approximately equal to morphine 10 mg IM**	No	80	55.6
	Yes	64	44.4
**Opioid should not be used in patients with a history of substance abuse**	No	77	53.5
	Yes	67	46.5
**Observation is part of the method used in surgical pain assessment**	No	49	34.0
	Yes	95	66.0
**The side effects of narcotics should be observed at least 20 min after administration**	No	50	34.7
	Yes	94	65.3
**If the source of pain is not known a pain drug should not be used during the pain evaluation period because this could mask the ability to correctly diagnose the cause of pain.**	No	41	28.5
	Yes	103	71.5
**Based on their cultural and spiritual beliefs Patients may think pain and suffering are necessary**	No	67	46.5
	Yes	77	53.5
**Patients should be encouraged to endure as much pain as possible before using an opioid.**	No	56	38.9
	Yes	88	61.1
**Pre-surgery injection such as anaesthesia is given for pain management**	No	59	41.0
	Yes	85	59.0
**Respiratory depression rarely occurs in patients who have been receiving stable doses of Opioids over a period of months.**	No	60	41.7
	Yes	84	58.3
**Rating scale ranging from (0) “no pain at all to (10) the worst pain” is essential to adopt in pain assessment**	No	65	41.1
	Yes	79	54.9
**If a patient sleeps with no movement postoperatively, this indicates that patient is not in pain**	No	67	46.5
	Yes	77	53.5

**Fig. 1:**
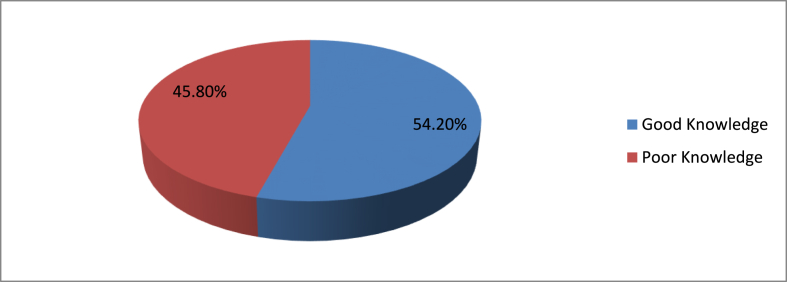
Knowledge of nurses toward post-operative pain management.

Among the total study participants, 78(54.2%) participants had good knowledge and 66(45.8%) participants had poor knowledge about postoperative pain management. (Fig. 1).

### 3.3 Attitude of nurses towards post-operative pain management

The mean Attitude of the respondents was 34.3 with ±3.8 standard deviation. Among study participants in the study 87(60.4%) had favorable Attitudes and 57(39.58%) unfavorable attitude. From all study participants 65(45.1%) believe patient with previous pain experience cannot increase current patients postoperative pain. 65(43.8%) of nurses as best one who can tell the feeling of patients pain (Table 3).

**Table 3:** Respondents attitude to Post-Operative pain Management.

**Name of the Variable**	Category	Frequency	**%**
**During postoperative period, patient may cry for many causes, not only from surgical wound.**	No	36	**25.0**
	Yes	108	**75.0**
**Pain should be assessed before & after administer pain drugs**	No	44	**30.6**
	Yes	100	**69.4**
**Each patient will have specific postoperative pain differently**	No	42	**29.2**
	Yes	102	**70.8**
**The patient should be advised to use non-drug techniques along with pain medication.**	No	38	**27.1**
	Yes	105	**72.9**
**Nurses' knowledge & understanding of postoperative pain management can make them be able to better manage the pain.**	No	37	**25.7**
	Yes	107	**74.3**
**Side effects of opioids should be observed at least (20–30) minutes after administration.**	No	48	**33.3**
	Yes	96	**66.7**
**During caring of patient, providing comfort and positioning may help to reduce muscle tension which in turn, can reduce pain.**	No	32	**22.2**
	Yes	112	**77.8**
**Appropriate assessment of pain is first priority for effective pain management in postoperative period.**	No	48	**33.3**
	Yes	96	**66.7**
**After operation, if the patient has severe pain, they may show abnormality in vital signs.**	No	52	**36.1**
	Yes	92	**63.9**
**After the initial recommended dose of opioid analgesics, subsequent doses should be adjusted according to the individual's patient's response.**	No	56	**38.9**
	Yes	88	**61.1**
**The most side effects of opioids (morphine) is respiratory depression.**	No	50	**34.7**
	Yes	94	**65.3**
**Previous pain experience cannot increase patient's current postoperative pain.**	No	65	**45.1**
	Yes	79	**54.9**
**The usual duration of action of IV morphine is 1**–**2 h.**	No	75	**52.1**
	Yes	69	**47.9**
**Pethidine are not generally recommended for treatment of children's postoperative pain.**	No	74	**51.4**
	Yes	70	**48.6**
**Nurse's role during postoperative pain management is to follow the doctors' order only.**	No	76	**52.8**
	Yes	68	**47.2**
**Nurse is the best one who can tell the feelings patient pain.**	No	63	**43.8**
	Yes	81	**56.3**
**Usually children perceive pain less than adult.**	No	67	**46.5**
	Yes	77	**53.5**
**Vital signs are always reliable indicators to assess the intensity of postoperative pain.**	No	52	**36.1**
	Yes	92	**63.9**
**If the source of pain is unknown; pain drug should not be used during the pain evaluation period, because this could mask the ability to correctly diagnose the cause of pain.**	No	48	**33.3**
	Yes	96	**66.7**
**Non drug interventions (eg. heat music, imaginary, touch is very effective for mild-moderate pain control but is rarely helpful for severe pain.**	No	46	**31.9**
	**Yes**	**98**	**68.1**

**Fig. 2:**
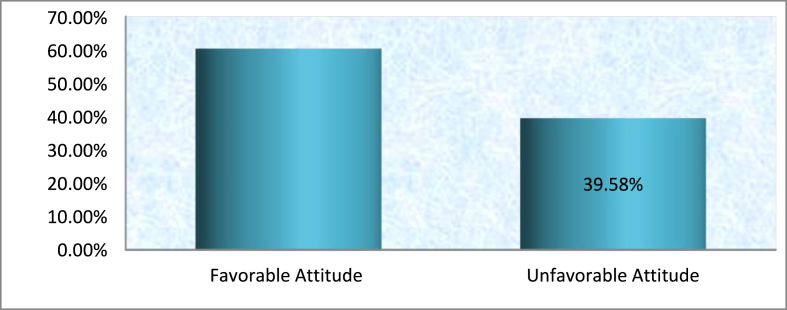
Attitude of nurses toward postoperative pain managements.

Among the 144 respondents that 83(57.6%) nurses had favorable attitudes and 61(42.4%) respondents, unfavorable attitudes toward pop management. (Fig. 2).

### 3.4 Respondents Practice of Nurses towards postoperative pain management

The mean score for the extent of practice of postoperative pain management was 20.04 with a standard deviation of 2.85. Study participants who scored but the mean were considered having poor practice, whereas participants who scored quite the norm were considered nurses to have good practice. Almost half the male group (55.7%) had good practice in postoperative pain management. 49(34%) of nurses do not observe patients vital sign (physiological) after surgery and 45(31.3%) of nurse's patient do not observe the side effect of pain medication such as morphine. From the current experience of nurses only 94(65.3%) use pain assessment tool and 48(33.3%) do not ever used. (Table 4).

**Table 4:** Respondents Practice of nurses towards postoperative pain management.

Name of the Variable	Category	Frequency	%percent
After surgery, do you suggest patient to tell the nurse when they are in pain?	No	43	**29.9**
	Yes	101	**70.1**
After surgery, do you observe behavioural change in-patient (such as awake, cry, limited body movement, with drawl, agitation, or deny to talk) in order to assess their pain?	No	34	**23.6**
	Yes	110	**76.4**
After surgery do you assess patients pain at least once a shift?	No	53	**36.8**
	Yes	91	**63.2**
After surgery, do you ask parents (attendant) to involve in assessing their patient's pain (such as asking patient if he/she have pain by using their familiar words &language?	No	49	**34**
	Yes	95	**66**
After surgery, do you observe physiological change in-patient (such as BP, HR, RR, T and oxygen saturation) in order to assess their pain?	No	40	**27.8**
	Yes	104	**72.2**
Do You administer pain medication to patient as ordered by doctors around the clock?	No	43	**29.9**
	Yes	101	**70.1**
Do You observe the side effects of pain medication (such as morphine) after giving it to the patient?	No	45	**31.3**
	Yes	99	**68.8**
After surgery, do you provided comfortable position to help relieve pain for patient?	No	60	**41.7**
	Yes	84	**58.3**
Do You ask & help patient to support the painful area during moving or coughing after surgery?	No	47	**32.6**
	Yes	97	**67.4**
After surgery, do you distract patient from pain by using entertainment?	No	58	**40.3**
	Yes	86	**59.7**
Do you use pain assessment tool	No	50	**34.7**
	Yes	94	**65.3**
Have you ever used pain assessment tools	No	48	**33.3**
	Yes	96	**66.7**

**Fig. 3:**
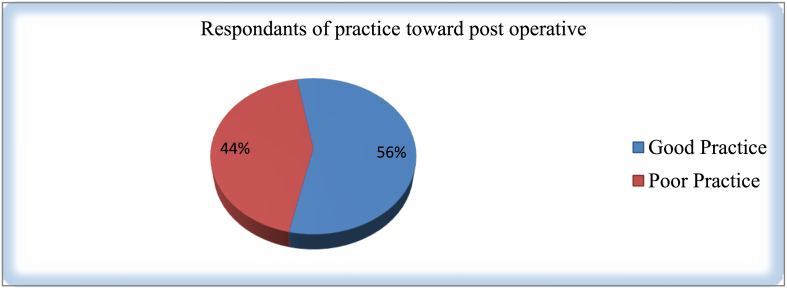
Respondents Practice of Nurses towards postoperative pain management.

The level of good practice in this study was 56% and participant with low level of practice were 46% (Fig. 3).

## 4 Discussions

Postoperative pain is a major public health problem in both developed and developing countries [9]. Nurses are important professionals who can improve the quality of pain management and provide nursing care to fully meet the needs of patients [11,29,30]. Therefore, this study aimed to evaluate the knowledge, attitudes, and practices of nurses in postoperative pain management.

According to our results, only 34 (23.6%) nurses were trained in postoperative pain management, indicating that less than half of the nurses were not trained in pain management, in contrast to a study by Zeb A et al. in which most nurses doing postoperative pain management are trained [12]. According to a study by Issa MR et al. training for nurses in pain management is effective in increasing knowledge and practice of pain management after surgery [17].

Regarding the pain management protocol in the participants' field of practice, 83 (57.6%) responded that no pain management protocols in the postoperative period, this were in contrast with the done by Adem AO et al. in which hospitals have protocols for pain management after surgery [8]. But the lack of a pain management protocol can be a major challenge for clinicians, as 80% of patients report postoperative pain, and 75% report moderate, severe, or possibly extreme pain. according to the study done by Dessie M et al. [15] and this is also further explained because, in more than 1/2 of the cases, the patients reported not having adequate pain control after their procedure according to Basak.S.et al. [10], this will lead to concerns about chronic pain. Therefore, hospitals should have guidelines and protocols for pain management according to a study by Ferrell et al. [25].

In our study, nurses' good knowledge of postoperative pain management was 78 (54.2%), comparable to a study in Ghana, Nigeria, and Ethiopia Aresi [13,14,16]. This may be because the majority of participants had comparable academic preparation and a lack of training in postoperative management. But the level of understanding of our study is low compared to the study of Beshir A et al. [27]. The reason for the inconsistent results between these studies may be due to differences in study location, nursing training level, socioeconomic level, and sample size. Of all study participants, 65 (45.1%) believed that patients who had experienced pain in the past could not increase the patient's postoperative pain, unlike the study by Manwere et al. [13]. Therefore, the current curriculum under the nursing program should be revised and an intensive and comprehensive program of pain management should be established as a requirement so that nursing students are also prepared. be good before graduation, depending on the field of study. Menlah A et al. [14]. This study showed that 87 (60.4%) nurses had a positive attitude toward pain management after surgery. This result is in agreement with the study conducted in Gondar [26], but our study is in contrast to the study conducted in WolaIta Sodo [28], in which nurses had an unfavorable attitude towards pain management after surgery. This could be due to different sample sizes, socio-demographic characteristics, and educational differences among nurses.

Regarding the current experience of nurses, only 94 (65.3%) used pain assessment tools and 48 (33.3%) never used them, comparable to a study by Man and para [25]. In which the pain assessment tool is inconsistent and inconsistent. But in nurse postoperative pain management, a valid and reliable pain assessment is critical for effective clinical care and research. Assessment of pain is essential to see the nature of the pain, whether pain management is appropriate, whether pain medication or a change in the dose of pain medication is needed, and whether other interventions are needed. Whether or not additional interventions, including specialist consultation to ensure optimal patient care, are appropriate, the nurse should have the appropriate knowledge, skills, and attitudes toward pain, and pain assessment and management. Must be supported by the most effective evidence available to prevent harm to patients [16,17,25]. In our study, the results showed 81 (56.25%) good practices. in the treatment of postoperative pain. Similar to a study conducted at Hawasa University Referral Hospital [19]. This could be explained by the similarity in the socio-demographic characteristics of the participants. Education level and training duration Our study results show that nurses practice pain management more after surgery than the study conducted at Arsizonale Hospital showed that 69 (47.9%)) have good practice in post-pain management [20]. This would be explained by differences in the classification of practice levels, sample size, and socio-demographic characteristics of the participants.

## 5 Conclusions

This study demonstrates inadequate training of nurses towards postoperative pain management, inadequate use of postoperative pain assessment tools, and nurses' overall knowledge, attitude, and practice toward postoperative pain management were around the mean score.

## Recommendation

We recommend recruiting nurses until hospitals are saturated enough to reduce nurses’ workload and available necessary materials like pain assessment tools, pain guidelines, and organizational protocols for pain assessment and management, We also recommend adjusting in-service training programs for all nurses to increase their knowledge, attitudes and practice toward POP management.

## Limitation

Single center study and our study was a descriptive cross-sectional study which limited to predictive factors for the low level of knowledge, attitude, and practice of nurses towards postoperative pain management.

## Implications for practice and future research

This study determines the level of knowledge; Nurses' attitudes and practices on postoperative pain management at Ambo reference hospital. Our research, which is exploratory and interpretive, creates a number of opportunities for future research, both in terms of theory development and concept validation. In fact, further research will be needed to refine and deepen our new findings. And it also suggests specific information about local guidelines, which guide training hospitals on POPM knowledge, attitudes and practices. In addition, it will provide stakeholder insight, providing background information for any researcher interested in conducting further research on POPM- and human-related issues. We believe that patients will receive quality care that will satisfy them personally. And will increase the result. with good pain recovery.

## Data statement

The data in this review is not sensitive in nature and is accessible in the public domain. The data is therefore available and not of a confidential nature.

## Ethical approval

Ethical approval was secured from Ambo University institutional review board.

## Sources of funding for your research

None.

## Author contribution

ZB, YB, WM and BG in Conceptualization, Validation, Data Cu ration, and Writing - Original Draft, Writing - Review & Editing.

## Research registration Unique Identifying number (UIN)

Name of the registry: Researchregistry.com
Unique Identifying number or registration ID: 7800
Hyperlink to your specific registration (must be publicly accessible and will be checked): Research Registry PDF AKP.pdf

## Guarantor

Zenebe Bekele.

## Provenance and peer review

**Not commissioned**, externally peer-reviewed.

## Declaration of competing interest

None.
